# Risk of Colorectal Cancer in Patients With Attention-Deficit Hyperactivity Disorder: A Nationwide, Population-Based Cohort Study

**DOI:** 10.3389/fpsyt.2021.537137

**Published:** 2021-02-05

**Authors:** Je-Ming Hu, Chia-Cheng Lee, Tzu-Chiao Lin, Chi-Hsiang Chung, Chao-Yang Chen, Pi-Kai Chang, Cheng-Wen Hsiao, Chien-An Sun, Nian-Sheng Tzeng, Wu-Chien Chien

**Affiliations:** ^1^Division of Colorectal Surgery, Department of surgery, Tri-Service General Hospital, School of Medicine, National Defense Medical Center, Taipei, Taiwan; ^2^Graduate Institute of Medical Sciences, National Defense Medical Center, Taipei, Taiwan; ^3^Medical Informatics Office, Tri-Service General Hospital, National Defense Medical Center, Taipei, Taiwan; ^4^Artificial Intelligence Center, Tri-Service General Hospital, National Defense Medical Center, Taipei, Taiwan; ^5^School of Public Health, National Defense Medical Center, Taipei, Taiwan; ^6^Department of Medical Research, Tri-Service General Hospital, National Defense Medical Center, Taipei, Taiwan; ^7^Taiwanese Injury Prevention and Safety Promotion Association, Taipei, Taiwan; ^8^Big Data Research Center, College of Medicine, Fu-Jen Catholic University, New Taipei City, Taiwan; ^9^Department of Public Health, College of Medicine, Fu-Jen Catholic University, New Taipei City, Taiwan; ^10^Department of Psychiatry, Tri-Service General Hospital, School of Medicine, National Defense Medical Center, Taipei, Taiwan; ^11^Student Counseling Center, National Defense Medical Center, Taipei, Taiwan; ^12^Graduate Institute of Life Sciences, National Defense Medical Center, Taipei, Taiwan

**Keywords:** attention-deficit hyperactivity disorder, colorectal cancer, retrospective cohort study, National Health Insurance Research Database, Longitudinal Health Insurance Database

## Abstract

**Background:** The association between attention-deficit hypersensitivity disorder (ADHD) and the risk of developing colorectal cancer (CRC) is, as yet, to be investigated, and thus, we have conducted this nationwide, cohort study to examine the association in patients from Taiwan.

**Methods:** In this study, 798 individuals with newly diagnosed ADHD and 2,394 (1:3) age-, gender-, and index year- matched controls without ADHD were enrolled, between 2000 and 2013, from the Longitudinal Health Insurance Database, a subset of the National Health Insurance Research Database in Taiwan. The cumulative incidence of CRC was assessed in each cohort by the Kaplan–Meier method. The multivariate Cox proportional hazards model was used to estimate the crude, and the adjusted hazards ratios (HRs) with 95% confidence intervals (CIs), was conducted to estimate the association between ADHD and CRC.

**Results:** The Kaplan–Meier analysis revealed that the cumulative incidence of CRC was significantly higher in patients with ADHD than in those without it (log rank test, *p* < 0.001). After adjustments for age, gender, comorbidities, and other covariates, the ADHD group was associated with an increased risk of CRC in comparison to the non-ADHD group (adjusted HR = 3.458, 95% CI = 1.640–7.293, *p* < 0.001). In addition, the usage of methylphenidate was not associated with the risk of developing CRC in patients with ADHD.

**Conclusion:** This retrospective cohort study depicts the evidence that ADHD was associated with the increased risk of CRC. Further studies are needed to confirm the association and the underlying mechanisms.

## Introduction

Colorectal cancer (CRC) is a major health challenge, representing the most common cancer and the third most frequent cause of cancer-related deaths in Taiwan (5,698 estimated deaths in 2012) (1). Several risk factors for CRC have been identified, such as smoking, dietary fat intake, obesity, and physical inactivity, and some studies have confirmed an excess of alcohol consumption may be a risk factor for a variety of cancers at the colorectal system (2). In addition, the average lifetime alcohol intakes were linearly associated with the risk of cancer and cancer-related death (3). Some recent evidence has suggested that specific gut microbiota contributes to the dysbiosis, mucosal integrity, immune deregulation, and immune-inflammatory alterations, and may contribute to the carcinogenesis (4). However, the mechanisms in the carcinogenesis of CRC are not, as yet, completely understood.

Attention-deficit/hyperactivity disorder (ADHD) is one of the most common pediatric neurodevelopmental and neurobehavioral disorders with a worldwide average prevalence estimated at 5% in children and 3.4–4.4% in adults, which results in attention deficit, hyperactivity, and increased impulsivity (5, 6), and up to 78% of ADHD patients have persisting symptoms into their adulthood (7). ADHD affects the health-related quality of life and leads to considerable school absences, family stress, and health care expenses (8–10). In addition, several studies have found that patients with ADHD are at risk of health problems, such as alcohol abuse (11, 12), cigarette smoking (13–15), and injury (16–18). Therefore, the identification, treatment, and management of ADHD are important and challenging in both children and adults. Psychostimulants are the first-line pharmacological treatment choice and have shown beneficial short-term efficacy (19). In addition, non-pharmacological treatments, such as parent training (20), behavioral interventions (20, 21), and neuro-feedback (22), could also be effective. However, several researchers have depicted that the varied relations between the ADHD medication methylphenidate (MPH) and cancer in human (23, 24) and animal studies (19, 25). However, the association between ADHD itself, with or without methylphenidate usages, and the risk of the development of cancer, is yet to be studied.

ADHD have bidirectional relations (26), for example, the increased gut microbiome predicts the function of the dopamine precursor synthesis between the ADHD cases and the controls (27), and the microbiota-gut-brain axis might play a role in the complex network of communication between the gut, intestinal microbiota, and the brain, by modulating the immune, gastrointestinal, and central nervous system functions (28). As aforementioned, gradual gut microbiota alterations are also linked to CRC in the initiation and progression during colorectal carcinogenesis (29, 30). Therefore, we hypothesize that there might be a link between ADHD and CRC, and we have thus conducted an explorative study to investigate the association between ADHD and CRC, by using a nationwide, population-based claims database, being Taiwan's National Health Insurance Research Database (NHIRD).

## Methods

### Data Source

The NHIRD was established in 1995, and as of June 2009, included contracts with 97% of the medical providers with ~23 million people in the program, or more than 99% of the entire population in Taiwan (31). The details of the program have been documented in previous studies (32–43). It contains comprehensive information, including the demographic data, inpatient and ambulatory claims, prescriptions, surgery, and other medical procedures claim records. The NHIRD, with multiple data sources, could represent a powerful research engine with enriched dimensions, and thereby serve as a guiding light for real-world evidence-based medicine in Taiwan (44). Diseases in the database are defined according to the International Classification of Disease, 9th Revision (ICD-9) codes.

### Study Subjects

**Figure 1:**
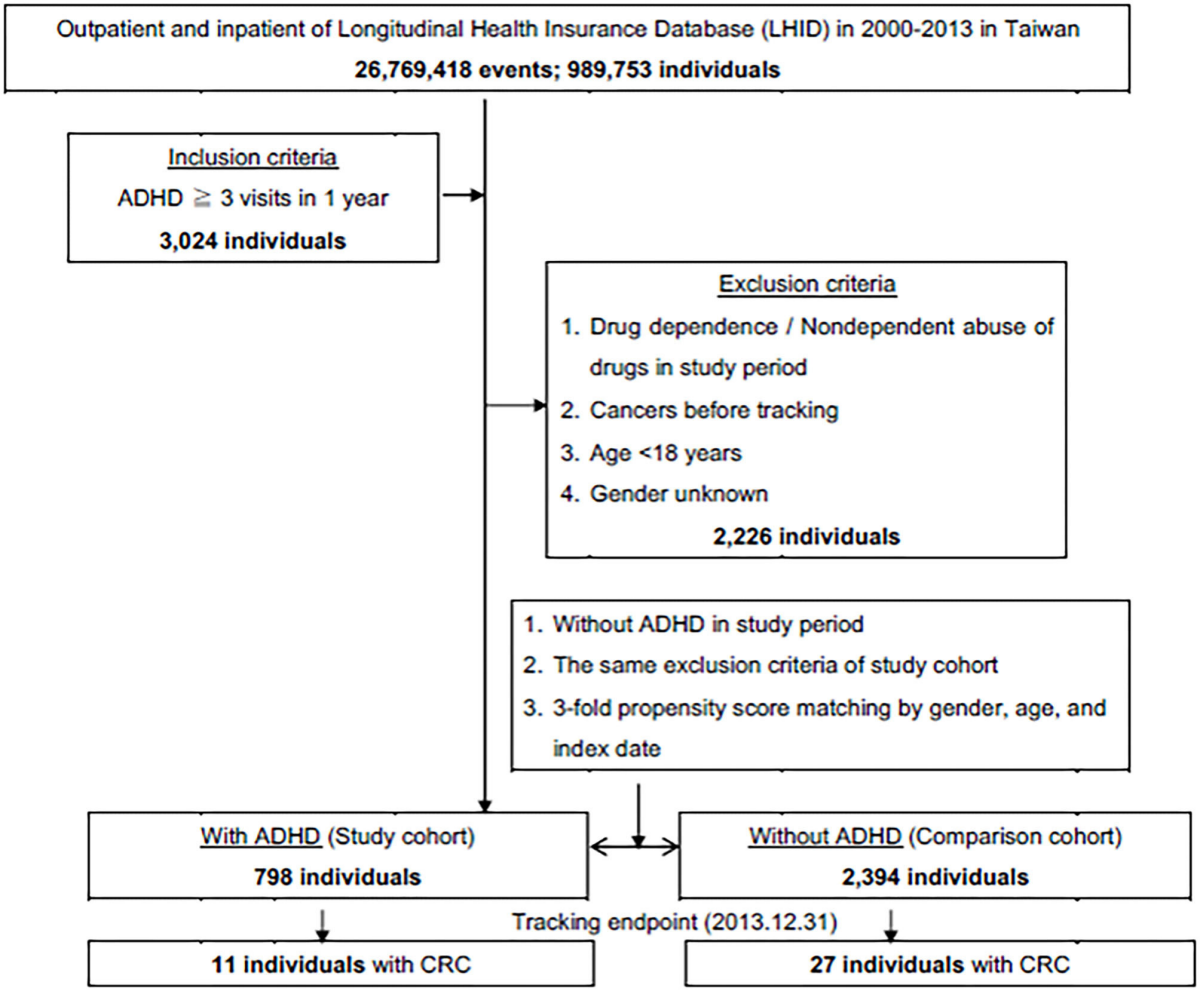
The flowchart of study sample selection from National Health Insurance Research Database in Taiwan.

This was a retrospective cohort study. Patients with ADHD between January 2000, and December 2013, were selected from the LHID and categorized according to the ICD as 314. All diagnoses of ADHD were made by board-certified specialists such as psychiatrists, pediatricians, neurologists, or physiatrists with specialty in child and adolescent development. The subject selection process is as presented in Figure 1. We also enrolled a 1:3 age-, gender-, and index year- matched control group of patients from the NHIRD, without a diagnosis of ADHD throughout the study period, as the unexposed group. We excluded patients who were younger than 18 years, data with unknown gender, drug abuse, and those diagnosed with cancer before the beginning of the follow-up from January 1, 2000.

### Outcome

We followed up both cohorts from January 1, 2000, until the date of CRC diagnosis (ICD-9-CM codes: 153, 153.0, 153.1, 153.2, 153.3, 153.6, 153.7, 153.8, 153.9,154, 154.0, 154.1, 154.2, 154.3, 154.8, and 159.0), withdrawal from the NHI, or the end of 2013.

### Covariates

The covariates included the age, gender, monthly insured premiums, comorbidities, locations, urbanization levels of residence, and the levels of hospitals for medical help. In the analysis, since the north Taiwan is the center of politics and economics, in the country. In addition, most of the healthcare resources, for example, 12 medical centers among total 23, located in the northern Taiwan. Therefore, the northern Taiwan was listed as the reference in our study for the locations.

### Comorbidities

We noticed the covariates that were potential confounders in the association between ADHD and CRC including age, gender, and the underlying chronic diseases related to the risk of developing CRC. Those chronic diseases, which were taken into account, included chronic obstructive pulmonary disease (COPD) (ICD-9-CM codes: 490, 491, 492, 493, 494, 495, and 496), diabetes mellitus (DM) (ICD-9-CM code: 250), coronary artery disease (CAD, ICD-9-CM codes: 410, 411, 412, 413, and 414), hypertension (HTN, ICD-9-CM codes:401, 402, 403, 404, and 405), hypercholesterolemia (ICD-9-CM codes: 272.0, 272.1, 272.2, and 272.4), alcohol-related diseases (ARD, ICD-9-CM codes: alcoholic liver disease, 571.0, 571.1, 571.2, and 571.3, and alcohol dependence, 303), peptic ulcer (ICD-9-CM codes: 531, 532, and 533); liver cirrhosis and chronic hepatitis (ICD-9-CM code: 571), inflammatory bowel disease (ICD-9-CM codes: 555 and 556), and psychiatric comorbidities such as oppositional defiant disorder (ODD, ICD-9-CM code: 313.81), conduct disorder (CD, ICD-9-CM code: 312), autism spectrum disorder (ASD, ICD-9-CM code: 299); tic disorder (ICD-9-CM code: 307.2); intellectual disabilities (ICD-9-CM codes: 317, 318, and 319); anxiety (ICD-9-CM code: 300); depression (ICD-9-CM codes: 296.2, 296.3, 300.4, and 311); and bipolar disorder (ICD-9-CM code: 296.0, 296.4-296.8). All the diagnosis of the psychiatric disorders was conducted by the board-certified psychiatrists, pediatricians, neurologists, and physiatrists.

### Statistical Analysis

We examined the descriptive statistics of the demographic characteristics and baseline comorbidities between the exposed and non-exposed cohorts by conducting chi-square tests or Student's-*t*-tests when appropriate. In addition, we used the Kaplan-Meier method to estimate the cumulative incidence of CRC in the study cohorts and performed the log-rank test to compare the difference between these two curves. We computed the hazard ratios (HRs) presented together with 95% confidence intervals (CIs) using the Cox proportional hazards models after adjusting for the potential confounders mentioned above. All the confounders, as covariates and comorbidities, including the psychiatric diagnoses, were calculated separately. All analyses were performed using the SAS version 9.4 (SAS Institute, Cary, NC) and the statistical significance was set to 0.05 in the 2-tailed tests.

## Results

### Sample Characteristics

A total of 3,192 patients were enrolled in this study, including 798 adult patients with ADHD and 2,394 patients in the non-ADHD control cohort. The age, gender, monthly insured premiums, comorbidities, locations and urbanization levels of residence, and the levels of hospitals for medical help are as summarized in Table 1. Most of the patients were in the age group of 20–49. The ADHD group tended to have higher rates of DM. HIN, ARD, and psychiatric comorbidities, such as ODD, CD, ASD, tics, intellectual disabilities, anxiety, depression, and bipolar disorders. In addition, the ADHD group tended to live in the North and the outlet islands of Taiwan, urbanization level 2, and seek their medical help from the regional hospitals.

**Table 1:** Characteristics of study at the baseline.

**Variables**	**Group total**		**With ADHD**		**Without ADHD**		***P***
	***n***	**%**	***n***	**%**	***n***	**%**	
Tot Total	3,192		798	25.00	2,394	75.00	
Gender							0.999
Male	2,312	72.43	578	72.43	1,734	72.43	
Female	880	27.57	220	27.57	660	27.57	
Age (years)	37.50 ± 20.17	36.89 ± 17.21	37.70 ± 21.06	0.326			
Age group (years)							0.999
20–49	2,328	72.93	582	72.93	1,746	72.93	
≧50	864	27.07	216	27.07	648	27.07	
Insured premium (NT$)							0.987
<18,000	2,722	85.28	682	85.46	2,040	85.21	
18,000–34,999	343	10.75	85	10.65	258	10.78	
≧35,000	127	3.98	31	3.88	96	4.01	
Comorbidity							
Chronic obstructive pulmonary disease	121	3.79	18	2.26	103	4.30	0.007
Diabetes mellitus	231	7.24	58	7.27	173	7.23	0.969
Coronary artery disease	123	3.85	21	2.63	102	4.26	0.043
Hypertension	250	7.83	66	8.27	184	7.69	0.595
Adult respiratory distress	68	2.13	27	3.38	41	1.71	0.007
Hypercholesterolemia	83	2.60	18	2.26	65	2.72	0.523
Peptic ulcer	156	4.89	19	2.38	137	5.72	<0.001
Liver cirrhosis and chronic hepatitis	141	4.42	11	1.38	130	5.43	<0.001
Inflammatory bowel disease	2	0.06	0	0.00	2	0.08	0.414
Oppositional defiant disorder	5	0.16	5	0.63	0	0.00	<0.001
Conduct disorder	32	1.00	32	4.01	0	0.00	<0.001
Autism spectrum disorder	44	1.38	43	5.39	1	0.04	<0.001
Tics	15	25.42	15	25.86	0	0.00	<0.001
Intellectual disabilities	82	2.57	80	10.03	2	0.08	<0.001
Anxiety	72	2.26	64	8.02	8	0.33	<0.001
Depression	138	4.32	133	16.67	5	0.21	<0.001
Bipolar disorders	97	3.04	93	11.65	4	0.17	<0.001
Location							<0.001
Northern Taiwan	1,334	41.79	402	50.38	932	38.93	
Middle Taiwan	813	25.47	171	21.43	642	26.82	
Southern Taiwan	852	26.69	185	23.18	667	27.86	
Eastern Taiwan	175	5.48	34	4.26	141	5.89	
Outlets islands	18	0.56	6	0.75	12	0.50	
Urbanization level							<0.001
1 (The highest)	1,059	33.18	245	30.70	814	34.00	
2	1,349	42.26	394	49.37	955	39.89	
3	258	8.08	41	5.14	217	9.06	
4 (The lowest)	526	16.48	118	14.79	408	17.04	
Level of care							<0.001
Hospital center	896	28.07	265	33.21	631	26.36	
Regional hospital	1,135	35.56	458	57.39	677	28.28	
Local hospital	1,161	36.37	75	9.40	1,086	45.36	

*ADHD, Attention-Deficit Hyperactivity Disorder*.

*P: Chi-square/Fisher exact test on category variables and t-test on continue variables*.

### Kaplan-Meier Model for the Cumulative Incidence of CRC

**Figure 2:**
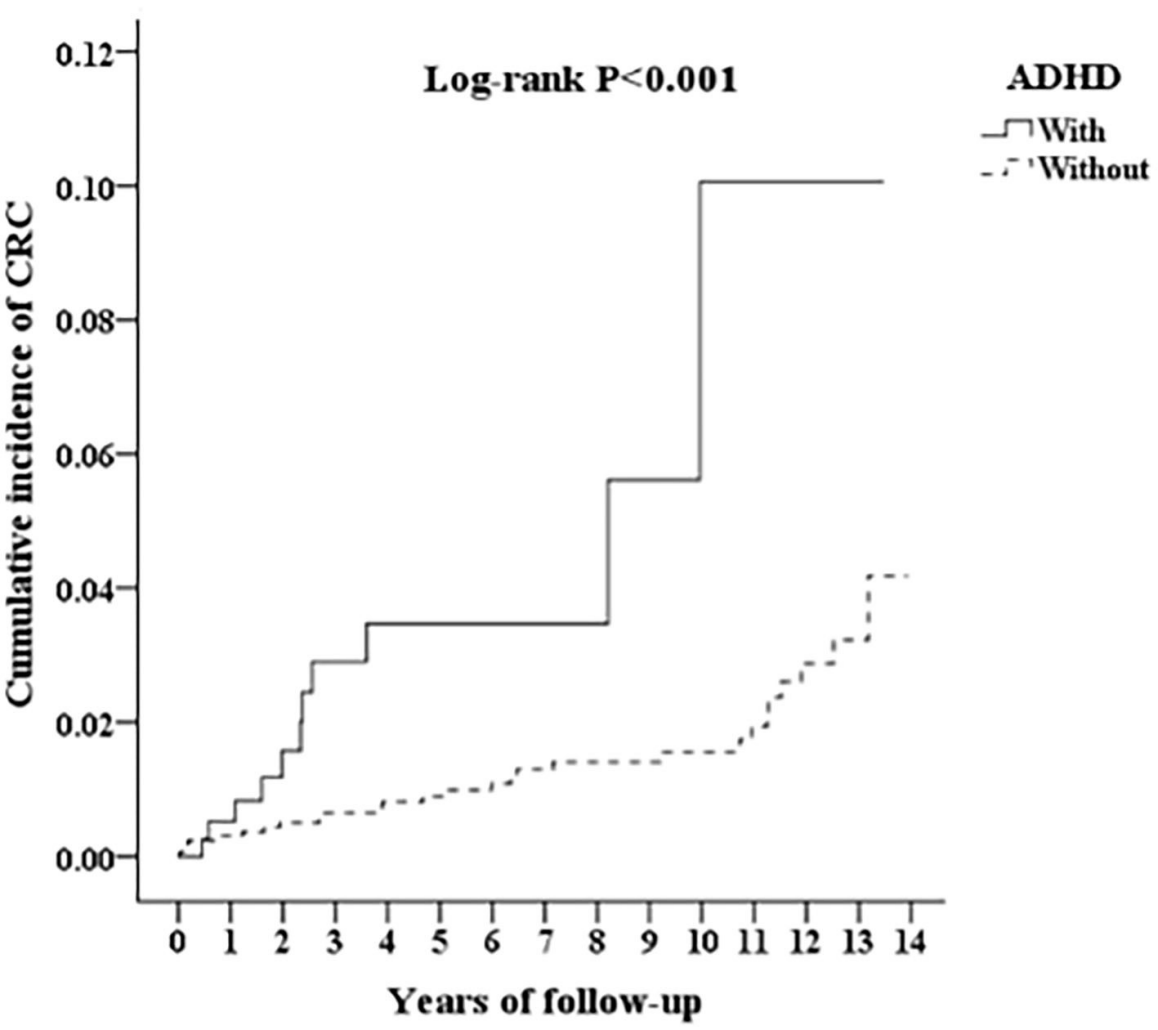
Kaplan-Meier for cumulative incidence of CRC among aged 18 and over stratified by ADHD with log-rank test.

The Kaplan-Meier analysis for the cumulative incidence of CRC in the ADHD and non-ADHD cohorts with the log-rank test revealed a significant difference over the 13-year follow-up period (*p* < 0.001) (Figure 2). In the 10th year of the study, the difference between the groups became significant (log-rank test *p* = 0.03).

### Hazard Ratios Analysis of CRC in the Patients With ADHD

Table 2 depicts that the multivariate Cox regression revealed a significantly higher risk of developing CRC in the ADHD cohort. The crude HR was 3.556 (95% CI = 1.700–7.480, *p* < 0.001), and after adjusting for age, gender, comorbidities, geographical area of residence, urbanization level of residence, and monthly income, the adjusted HR was 3.458 (95% CI = 1.640–7.293; *p* < 0.001). For the participants older than 50 years, in both the ADHD and non-ADHD cohort, the risk of CRC was 6.887 (95% CI = 2.371–20.005, *p* < 0.001), in comparison to the subgroup aged from 20 to 49. In contrast, the ADHD group was uniformly associated with an increased risk of CRC for all the factors.

**Table 2:** Factors of Colorectal cancer by using Cox regression.

**Variables**	**Crude HR**	**95% CI**		***P***	**Adjusted HR**	**95% CI**		***P***
**Attention deficit- hyperactivity disorder**								
Without	Reference			0.001	Reference			
With	3.556	1.700	7.480	0.001	3.458	1.640	7.293	0.001
**Gender**								
Male	1.530	1.278	2.010	0.154	1.070	0.347	1.294	0.233
Female	Reference				Reference			
**Age group (years**)								
**20–49**	Reference				Reference			
**≧50**	7.052	2.498	19.913	<0.001	6.887	2.371	20.005	<0.001
**Insured premium (NT$)**								
<18,000	Reference				Reference			
18,000–34,999	0.000	–	–	0.604	0.000	–	–	0.989
≧35,000	0.000	–	–	0.737	0.000	–	–	0.995
**Location**								
Northern Taiwan	Reference				Multi-collinearity with urbanization level			
Middle Taiwan	0.411	0.165	1.023	0.056	Multi-collinearity with urbanization level			
Southern Taiwan	0.690	0.331	1.414	0.324	Multi-collinearity with urbanization level			
Eastern Taiwan	0.257	0.034	1.916	0.185	Multi-collinearity with urbanization level			
Outlets islands	0.000	–	–	0.979	Multi-collinearity with urbanization level			
**Urbanization level**								
1 (The highest)	4.155	0.944	18.287	0.060	4.298	0.712	25.944	0.129
2	3.754	0.627	22.467	0.147	3.227	0.712	14.632	0.126
3	3.927	0.915	16.683	0.660	3.413	0.707	16.466	0.112
4 (The lowest)	Reference				Reference			
**Level of care**								
Hospital center	2.748	1.025	7.363	0.044	1.896	0.645	5.578	0.245
Regional hospital	1.504	0.541	4.180	0.434	1.174	0.412	3.344	0.764
Local hospital	Reference				Reference			

*HR, hazard ratio; CI, confidence interval. Adjusted HR, adjusted for covariates in Table 1. NT$, New Taiwan Dollars*.

*P: Chi-square/Fisher exact test on category variables and t-test on continue variables*.

### Subgroup Analysis of CRC in the Patients With ADHD

**Figure 3:**
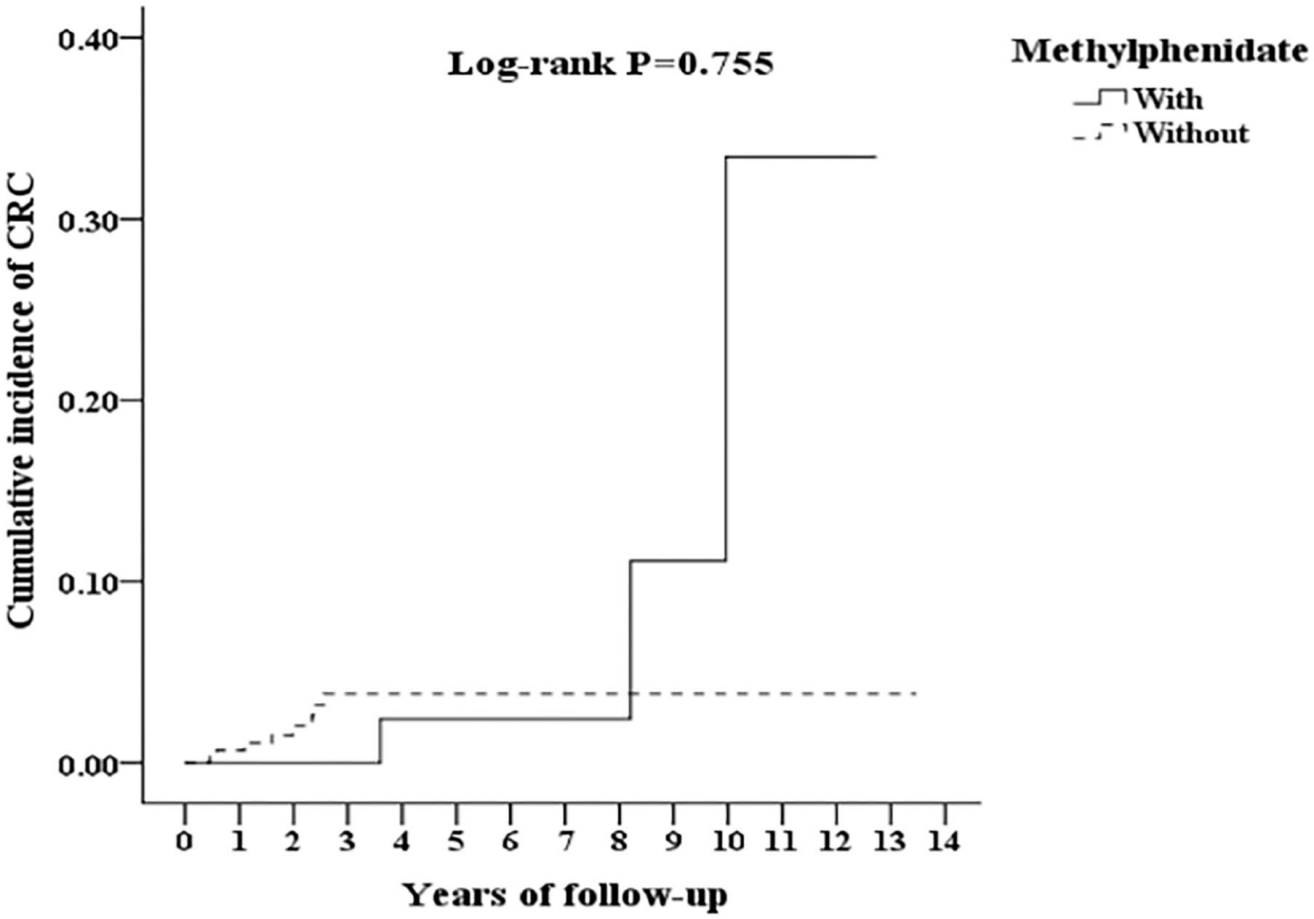
Kaplan-Meier for cumulative incidence of CRC among ADHD aged 18 and over stratified by Methylphenidate with log-rank test.

In Table 3, after the stratification according to age, gender, and covariates, the risk of development of CRC was higher in the ADHD group than the control group. We found that adults with ADHD were associated with an increased risk of developing CRC no matter the age group, gender, and with or without comorbidities, such as COPD, DM, CAD, and HTN, in comparison with the controls. Besides, the patients in the ADHD group without comorbidities similar to ARD, hypercholesterolemia, peptic disease, liver cirrhosis, and chronic hepatitis, ODD/CD, ASD, tics, intellectual disabilities, anxiety, and bipolar disorder, were associated with an increased risk of developing CRC when compared to the controls. However, we did not find any association between the usage of MPH and the risk of developing CRC in the patients with ADHD (Figure 3).

**Table 3:** Factors of Colorectal cancer stratified by variables listed in the table by using Cox regression.

**ADHD (With vs. Without)**	**Adjusted HR**	**95% CI**	**95% CI**	***P***
**Stratified**				
Total	3.458	1.640	7.293	0.001
**Gender**				
Male	3.930	1.608	7.149	<0.001
Female	3.241	1.537	6.834	0.001
**Age group (years)**				
20–49	2.121	1.145	4.997	0.003
≧50	3.529	2.398	7.561	0.001
**Insured premium (NT$)**				
<18,000	3.458	1.640	7.293	0.001
18,000–34,999	–	–	–	–
≧35,000	–	–	–	–
**Urbanization level**				
1 (The highest)	4.984	2.364	9.512	<0.001
2	2.915	1.382	6.147	<0.001
3	3.866	1.833	8.153	<0.001
4 (The lowest)	2.151	1.035	4.345	0.034
**Level of care**				
Hospital center	3.661	1.736	7.721	<0.001
Regional hospital	3.531	1.675	7.470	0.001
Local hospital	2.413	1.144	5.008	0.001

*ADHD, Attention-Deficit Hyperactivity Disorder; Adjusted HR, Adjusted Hazard ratio; adjusted for the covariates in the Table 1. NT$, New Taiwan Dollars*.

*P: Chi-square/Fisher exact test on category variables and t-test on continue variables*.

## Discussion

### Association Between ADHD and the Risk of CRC

In this study, we examined the association between ADHD and the risk of CRC. After adjusting the covariates, the adjusted HR was 3.458 for the ADHD group (95% CI = 1.640–7.293; *p* < 0.001), when compared with the non-ADHD control group. The Kaplan-Meier analysis revealed that the study subjects had a significantly higher 14-year cumulative incidence of ADHD than the controls. Furthermore, the ADHD-cohorts older than 50 years had a nearly 6.9-fold increased risk of CRC (adjusted HR: 6.887 [95% CI: 2.371–20.005; *p* < 0.001]). This study revealed that patients with ADHD had a nearly 3.5-fold risk of CRC, and this report could be a reminder for the clinicians who care the patients of ADHD in the follow-up. To the best of our knowledge, this is the first nationwide, population-based cohort on the topic of the association between ADHD and the risk of developing CRC.

### Comparison of This Study to Previous Literatures

Several previous studies have shown that the overall psychiatric disorders were associated with the increased risk of cancer (45, 46). Additionally, some studies have found that depression (47), eating disorders (48), and posttraumatic disorder (49) are associated with the increased risk of different types of cancer, but the risk of cancer in schizophrenia and bipolar disorder varied with gender and age (50). Prior studies have revealed that psychotropic medications, such as antidepressants, were associated with several cancers, such as breast cancer (51, 52), nasopharyngeal cancer (53), and Hodgkin's lymphoma (54). The usage of MPH and other drugs for ADHD have suggested an increase in the risk of developing cancers, especially in vulnerable elderly patients and in high-dosage groups (24, 25), but there is some controversy in this association (24, 55).

### Possible Mechanisms for the Increased Risk of CRC in Patients With ADHD

The underlying mechanisms of the association between ADHD and CRC remain unknown. We hypothesize several factors for this association: Owing to the lifestyles, behavioral problems, and comorbidities related to ADHD (16, 18, 57–59), they are at risk of alcohol abuse (11, 12), cigarette smoking (13–15), and obesity (60), and these problems are, in turn, the risk factors of CRC (61–63). In addition, the socio-economic disadvantages might well-contribute to the risk of ADHD (64, 65) and CRC (66, 67). Our results from the subgroup analyses showed that the urbanization level-specific ADHD in comparison to the HR of CRC was significant for patients living in the residence of urbanization level 1, having higher monthly insurance premiums, and visiting hospital centers. Moreover, patients living in higher urbanization levels had a greater risk of CRC. However, further studies are needed to clarify the role of the interactions among the lifestyles, behavioral problems, and socio-economic disadvantages in the risk of developing CRC in the ADHD patients.

Furthermore, ADHD and CRC share several common links such as gut microbiota, hypothalamic-pituitary-adrenal axis, chemokines, cytokines, short-chain fatty acids, autonomic nervous system, and enteric nervous systems (28). Several animal model studies have suggested that gut microbiota may be involved in the development of brain-related disorders (68). The gut microbiota could influence the reward centers of the brain with dopamine (DA) that have been found in people with ADHD (27).

Several studies have found a higher presence of asthma (69), eczema, and rhinitis (70, 71) in patients with ADHD. Studies also identified a higher prevalence of autoimmune diseases, such as thyrotoxicosis, type 1 diabetes, autoimmune hepatitis, psoriasis, and ankylosing spondylitis in ADHD patients (72). Thus, inflammation might play an important role in the mechanism in ADHD. Moreover, the connection between inflammation and CRC tumorigenesis is well-established (73, 74). Therefore, inflammation might be a common link between ADHD and CRC.

Prior studies have found that peripheral DA plays an important role of the tumor's immunity (75), and dopamine significantly enhances the efficacies of the commonly used anticancer drugs (76). In addition, one of the potential underlying mechanisms is the imbalance of the dopaminergic system (77, 78). Therefore, further studies are needed to investigate as to whether there is a common link between ADHD and CRC.

There was a possible pathophysiology role correlation between MPH treatment and cancer, for an elevated incidence of chromosomal anomalies related to MPH (24). However, the present study does not support the association between MPH and the risk of CRC. As aforementioned, inflammation might play a role as the common link between ADHD and CRC. One finding suggests that MPH could down-regulate the inflammatory markers, and thus might be one of the reasons that MPH is noted associated with CRC (56). This discrepancy between these two studies warrants a further study.

### Strengths

Our study has several strengths. First, it was conducted by using the NHIRD, a claims database widely used for academic research, was retrieved from the NHI program, a universal, single-payer health insurance system, which comprises comprehensive information, including the demographic data, dates of medical visits, and medical services (79). Second, in this database with a high coverage of people in Taiwan, which is a large, nationwide, and population-based sample, that avoids the selection and participation biases (79). Third, the criteria of ADHD and CRC were defined according to ICD-9-CM, which were monitored and strictly evaluated by the NHI Administration for the reimbursement agency, and we could have also adjusted the covariates from this nationwide database to estimate the association between ADHD and the risk of developing CRC (80).

### Limitations

We are aware of the limitations of our study. First, the diagnoses of ADHD and CRC were based on the diagnostic codes recorded manually by the physicians into the National Health Insurance (NHI) claim database system; therefore, some registry bias may have been involved in the calculation of the CRC risk. In addition, the family history of colorectal cancer is present, up to one third of patients (81). The lack of the information of the genetic-family risk factor in this claims database study is an important limitation. Second, the socio-economic status, which is an important contributory factor to ADHD and CRC, as aforementioned, could only be represented by the monthly insurance premiums, urbanization levels and the location of the residences, and the hospitals of the patients seeking medical help. Third, the severity of ADHD and CRC and the functional status of the ADHD patients were not evaluated in the NHIRD.

## Conclusion

This retrospective cohort study provided evidence of a nearly 3.5-fold increased risk of CRC in ADHD. The results of this study could serve as a reminder for the clinicians who care for the patients of ADHD in the follow-up. Further prospective studies are necessary for confirming our findings, we therefore recommend meticulous evaluation and aggressive risk reduction for CRC for the patients with ADHD.

## Data Availability Statement

The datasets analyzed in this article are not publicly available. Requests to access the datasets should be directed to Data are available from the National Health Insurance Research Database (NHIRD) published by the Taiwan National Health Insurance (NHI) Administration. Due to legal restrictions imposed by the government of Taiwan in relation to the “Personal Information Protection Act”, data cannot be made publicly available. Requests for data can be sent as a formal proposal to the NHIRD (https://nhird.nhri.org.tw/en/index.html).

## Ethics Statement

The studies involving human participants were reviewed and approved by the Institutional Review Board of the Tri-Service General hospital approved the protocol for this study (TSGHIRB NO. 2-106-05-029). Written informed consent for participation was not required for this study in accordance with the national legislation and the institutional requirements.

## Author Contributions

J-MH, C-CL, N-ST, and W-CC conceived, planned, and conducted this study. C-CL, J-MH, N-ST, C-HC, and W-CC contributed to the data analysis and interpretation. J-MH, T-CL, C-HC, C-YC, P-KC, C-AS, and C-WH contributed to this data interpretation. C-CL wrote the first draft. J-MH has played major role, in this revision, in the concept, data interpretation, data analysis, and the re-writing of this manuscript. N-ST and W-CC conducted the critical revisions of this article. All authors approved this manuscript.

## Conflict of Interest

The authors declare that the research was conducted in the absence of any commercial or financial relationships that could be construed as a potential conflict of interest.
